# Usage of the National Cancer Institute Cancer Research Data Commons by Researchers: A Scoping Review of the Literature

**DOI:** 10.1200/CCI.24.00116

**Published:** 2024-11-13

**Authors:** Zhaoyi Chen, Erika Kim, Tanja Davidsen, Jill S. Barnholtz-Sloan

**Affiliations:** ^1^ Informatics and Data Science Program, Center for Biomedical Informatics and Information Technology, National Cancer Institute, Rockville, MD; ^2^ Office of Data Science and Strategy, National Institutes of Health, Bethesda, MD; ^3^ Division of Cancer Epidemiology and Genetics, National Cancer Institute, Rockville, MD

## Abstract

**PURPOSE:**

Over the past decade, significant surges in cancer data of all types have happened. To promote sharing and use of these rich data, the National Cancer Institute's Cancer Research Data Commons (CRDC) was developed as a cloud-based infrastructure that provides a large, comprehensive, and expanding collection of cancer data with tools for analysis. We conducted this scoping review of articles to provide an overview of how CRDC resources are being used by cancer researchers.

**METHODS:**

A thorough literature search was conducted to identify all relevant publications. We included publications that directly cited CRDC resources to specifically examine the impact and contributions of CRDC by itself. We summarized the distributions and trends of how CRDC components were used by the research community and discussed current research gaps and future opportunities.

**RESULTS:**

In terms of CRDC resources used by the research community, encouraging trends in utilization were observed, suggesting that CRDC has become an important building block for fostering a wide range of cancer research. We also noted a few areas where current applications are rather lacking and provided insights on how improvements can be made by CRDC and research community.

**CONCLUSION:**

CRDC, as the foundation of a National Cancer Data Ecosystem, will continue empowering the research community to effectively leverage cancer-related data, uncover novel strategies, and address the needs of patients with cancer, ultimately combatting this disease more effectively.

## Teaser

Scoping review shows NCI's CRDC is a key tool for advancing cancer research, with room for growth.

## INTRODUCTION

Over the past decade, cancer research has experienced an explosion in cutting-edge technologies and breakthroughs, which led to significant surges in cancer data of all types. These rich data sets provide researchers with opportunities to derive knowledge to combat cancer. For example, genomics data from genome sequencing and profiling have been widely used to identify new biomarkers and molecular targets for targeted therapies,^1-3^ whereas imaging data have been used to develop screening and early detections.^4-6^ With recent advancements in machine learning models and artificial intelligence, structured data and unstructured clinical data from electronic health record systems have been increasingly used to develop risk models and to examine real-world patient outcomes.^7-10^

To support future cancer research and promote data sharing, National Cancer Institute (NCI) has established a series of data commons and cloud resources, collectively known as the Cancer Research Data Commons (CRDC).^11^ Today, the CRDC has evolved into a cloud-based platform that provides access to a variety of data sets, tools, and applications. Over the years, new tools have been gradually added under the umbrella of CRDC, including cloud-based computing platforms, data standards and definitions, and a wide range of resources and data services.

We have recently described the details of all the CRDC resources in a series of manuscripts, which include data commons, core infrastructure services, and cloud resources. These papers highlighted CRDC's current state and plans for future developments.^12-15^ In terms of data, there are six Data Commons available within CRDC: Genomic Data Commons (GDC), Proteomic Data Commons, Imaging Data Commons (IDC), Integrated Canine Data Commons (ICDC), Clinical Trial Data Commons, and Cancer Data Service.^14^ Collaborating with leading biomedical analytical partners, three cloud computing platforms are currently supported by CRDC: Broad Institute FireCloud, ISB Cancer Gateway in the Cloud (ISB-CGC), and Seven Bridges Cancer Genomics Cloud (SB-CGC).^13^ These Cloud Resources provide access to hundreds of analysis tools and allow researchers to create custom analytic workflows in an easy-to-use, cloud-based environment, allowing analysis of CRDC raw and derived data without the need to download or move large data sets. Finally, there are several essential services provided by CRDC aiming to improve the transparency and searchability of CRDC data for downstream analysis, such as Cancer Data Aggregator (CDA), Data Commons Framework, and Data Standards Services (DSS).^12^

Since its launch, the CRDC has had a significant impact on cancer research over the past 10 years. Currently, the CRDC holds more than 9.4 petabytes data from 354 studies. Each year, more than 82,000 users are accessing and using CRDC resources. These numbers highlight the increasing demand for access to quality data for cancer research. In this scoping review, we are taking a deeper look at published studies using these resources to understand how they are currently being leveraged by researchers. Doing so allows us to gain a better understanding of the value of these resources from users' point of view and provides insights into areas for future improvements.

## METHODS

After the recommendation from the Preferred Reporting Items for Systematic Reviews and Mata-Analyses extension for Scoping Reviews (PRISMA-ScR),^16^ a thorough literature search was conducted in December 2023 to identify all relevant publications until December 31, 2023. Our main selection criteria are to include only the publications that directly cited CRDC resources. This approach allows us to specifically examine the impacts and contributions of CRDC by itself, and by focusing on these studies, we will be able to gain insights into the different ways in which researchers are leveraging the CRDC and evaluate the effectiveness of CRDC in supporting research endeavors. Hence, search terms were constructed on the basis of each individual component (ie, data commons, infrastructure, and cloud resources) of the CRDC. In addition, we intentionally did not use the names of individual data sets/studies as part of our search strategy (eg, terms like “The Cancer Genome Atlas” or “TCGA” were not part of our search although the TCGA data can be accessed as part of the GDC), and we excluded papers that did not directly use CRDC resources (eg, access data from other sources). The final search keywords we used are listed in Table 1.

**TABLE 1.** Keywords Used for Literature Search

Category	Keywords	Relationship in Search
Overall	Cancer Research Data Commons	Or
Data commons	Cancer Data Service, Clinical Trial Data Commons, Genomic Data Commons, Imaging Data Commons, Integrated Canine Data Commons, Proteomic Data Commons	Or
Infrastructure	Cancer Data Aggregator, Data Commons Framework, Data Standard Services	Or
Cloud resources	Broad Institute FireCloud, ISB Cancer Gateway in the Cloud, Seven Bridges Cancer Genomics Cloud	Or

**FIG 1.**
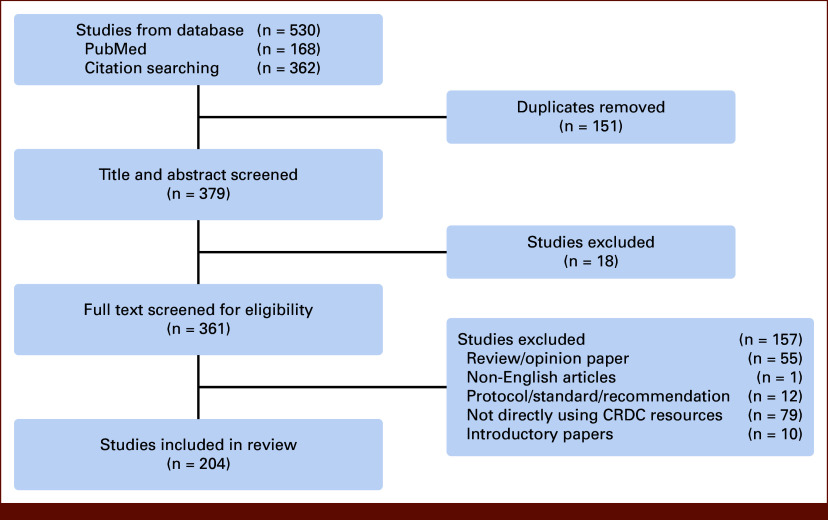
Flow diagram showing the selection of publications. CRDC, Cancer Research Data Commons.

To exhaust all relevant articles, an iterative literature search strategy was used; a total of two rounds of the search were conducted in PubMed (Fig 1). The first round of literature search was conducted using the search terms described above. We then excluded introductory papers from original developers; review and opinion papers; protocol, standard, or recommendations; and articles not directly using CRDC resources. The introductory papers identified in this round, although excluded from our review, were then used in the second round of literature search using a citation search approach: all papers that cited these introductory papers were identified and included using the cited by function in PubMed. After the second round of literature search, we followed the standard review process to screen the included publications and extract relevant information. A data-charting form was developed to determine which variables to extract. Data from eligible studies were collected using a standardized data extraction form. We extracted data on article characteristics (eg, country of origin, publication year, funder) and study characteristics (eg, type of analysis, research question, resources used). To comply with PRISMA-ScR, a completed checklist is provided in the Data Supplement (Table S1).

## RESULTS

Overall, 379 papers were identified after removal of duplicates. After abstract and full text screening, a total of 204 papers were included. For all included papers, information on how CRDC resources were used was extracted and summarized. A full list of included papers is provided in the Data Supplement (Table S2). Details on the article selection process is shown in Figure 1, including the number of publications identified, included, and excluded and the reasons for exclusions. In addition, among the 10 introductory papers identified, five described the GDC, four of them described Cloud Resources (eg, SB-CGC, ISB-CGC), and one paper described the IDC. A full list of these introductory papers is shown in the Data Supplement (Table S3).

**FIG 2.**
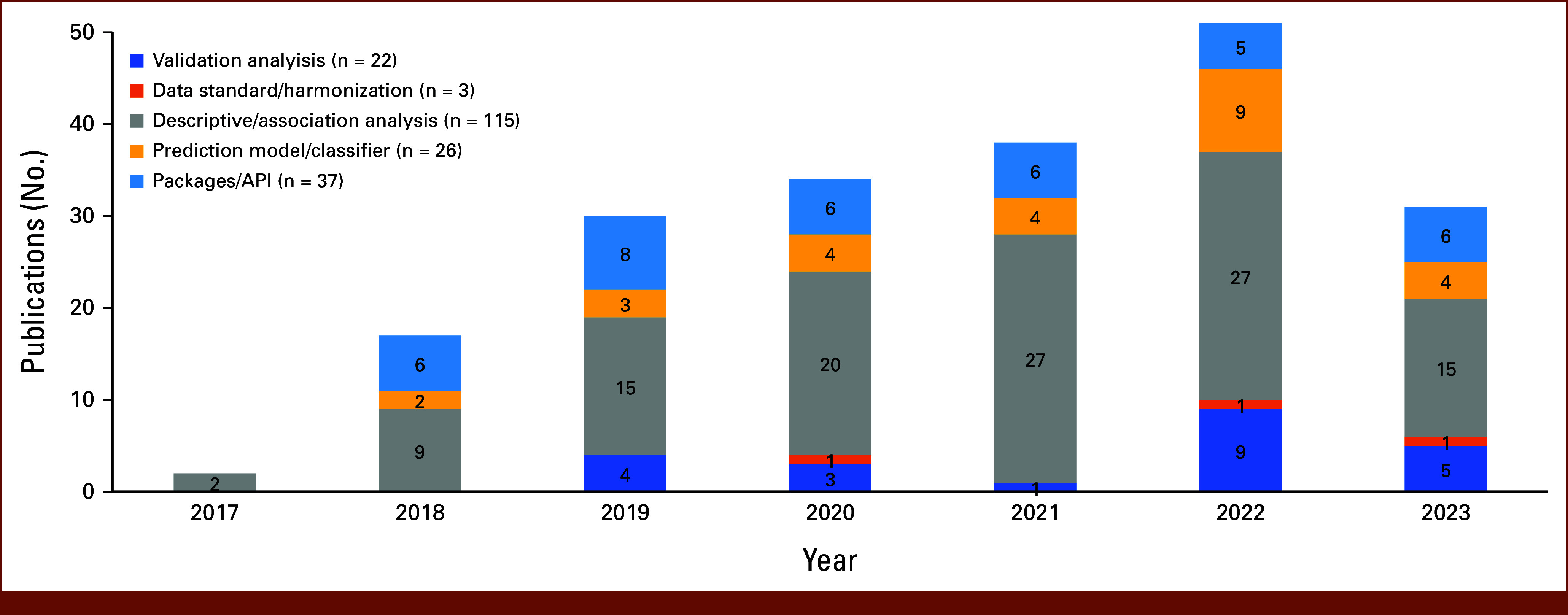
Number of included publications by year and by type of analysis (n = 204 total publications). API, application programming interface.

To understand the scope of impact of the CRDC on cancer research, we first examined the number of papers by year (Fig 2). As CRDC grows over the years, adding new data sets and tools to the ecosystem, a steady increase in the number of publications has been observed. Figure 2 also breaks down the number of papers by the types of research questions being answered. In addition to the increasing number of publications, we observed increasingly diverse ways of utilization: most of the early studies were descriptive or association analysis, whereas a wider range of research tasks were seen from the more recent publications.

Overall, more than half (n = 115, 56.4%) of the included studies are descriptive analyses that examined the associations between biomarkers and cancer risks or outcomes. A good proportion (n = 63, 30.9%) of studies also developed prediction models or analytical packages. Moreover, tools from most of these studies (n = 45) were made available by the research team either on public platforms (eg, GitHub) or through CDRC platforms (eg, SB-CGC). For example, Yu et al^17^ developed and released a fast, memory-efficient indexing structure to query large RNA-seq data sets, and it showed great performance and efficiency in TCGA Pan-Cancer data sets. More recently, Xiao et al^18^ developed an application to facilitate the generation of BioCompute Object from both plaintext workflow metadata and workflow written in the Common Workflow Language. The application can be directly accessed in the SB-CGC and incorporated with users' workflow.

In addition, we identified 22 articles that were validation studies using CRDC resources. Some of them compared findings from other cohorts (usually primary collected data) with data from GDC. For example, Dotson et al^19^ examined the gene expression from RNA samples extracted from a single needle pass and compared it with the corresponding TCGA data sets, and consistent transcriptomic results and differential expression patterns were observed. Parolia et al^20^ identified three structural classes of *FOXA1* alterations in a cohort of patients with advanced prostate cancer and then used TCGA data to confirm the classifications. In other publications, the authors developed prediction models and validated the performances using data from CRDC Data Commons. For example, Xia et al^21^ cross-validated a drug response prediction model in five different data sets, including ones from GDC. Here, the authors trained prediction models using each data set and examined the performance of the models on other data sets. They found that the performance of the models varied largely depending on the data sets, highlighting the need for external validation and model calibration.

In terms of data source, we found that GDC is the most popular resource being accessed in these publications. A total of 196 publications used GDC to obtain genomics data for further analysis. We also noticed that certain data sets were used significantly more frequently than other data sets. For example, TCGA data have been used as the main (or only) data source in more than 80% (180 papers, 88.2%) of the publications. As a landmark cancer genomic program, TCGA has characterized over 20,000 primary cancers and matched normal samples for 33 cancer types. The publicly accessible comprehensive molecular characterization from a large study population makes it one of the most popular sources.^22,23^

**FIG 3.**
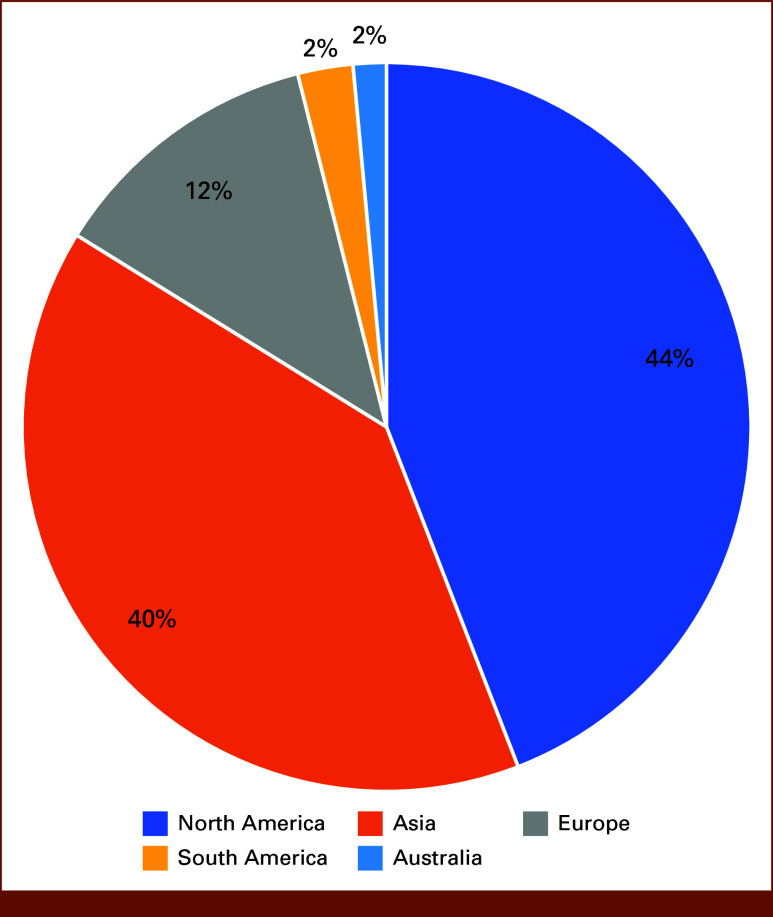
Number of publications by region (n = 204 total publications).

Furthermore, CRDC has also made a significant global impact. Among the included 204 publications, more than half (56%) of them were conducted by research teams outside North America (Fig 3). The importance of sharing data lies in its capacity to accelerate discoveries and innovations by allowing researchers worldwide to build upon existing knowledge. Collaboration, both within and across borders, leverages diverse expertise and resources, leading to more comprehensive and robust research outcomes. As our observation suggests, the CRDC has made a significant contribution in these aspects by serving as a valuable resource for researchers worldwide. Additional efforts have also been made to promote data interoperability and reuse. The CRDC is currently adapting standards like Global Alliance for Genomics and Health Data Connect and Fast Health Interoperability Resources to facilitate standardized metadata for researchers to find data sets and cohorts of interest.^24,25^

In terms of disease domain, these publications covered a wide range of different types of cancers, with more than a fourth (26.8%) being pan-cancer analyses. Research questions being addressed in these analyses include the identification of germline variants associated with patient outcomes, identification of gene fusions associated with cancer risk, characterization of cancer microbiomes, and more.^26-30^ Data deposited in CRDC are particularly suitable for pan-cancer analysis because CRDC data cover many types of cancer with larger sample sizes. We also saw several papers using CRDC resources in diseases other than cancer. For example, using the SB-CGC platform, Lim et al developed and shared a cloud workflow for mutation profiling of SARS-CoV-2. Their workflow was shown to be able to differentiate SARS-CoV-2 cases with high accuracy.^31-33^ Lehrer et al examined the relationship between genes implicated in Alzheimer's disease and genes implicated in prostate cancer and found that alterations in prostate cancer gene speckle-type POZ protein significantly co-occurred with alterations in Alzheimer's disease gene bridging integrator-1, which may help examining the increased risk of Alzheimer's disease and cognitive impairment in patients with prostate cancer treated with androgen deprivation therapy.^34,35^ Moreover, Caro-Vegas et al^36^ compared the exome sequence of breast and lung cancers between women living with HIV and a HIV-negative cohort and found that those who have HIV have significantly higher tumor mutational burden. These studies showed that CRDC resources, particularly Data Commons and Cloud Resources, are powerful and flexible for understanding other common diseases outside of cancer.

## DISCUSSION

In this review, we examined how CRDC resources were used by the research community. Encouraging trends in utilization were observed, suggesting that CRDC has become an important building block for fostering a wide range of cancer research. Over time, the number and diversity of publications have increased. In addition, while the CRDC is an NCI effort based in the United States, over half of the publications were from authors outside North America. The data are being leveraged for cross cancer comparisons and comparisons between cancer and other common diseases. We also noted that many studies have made efforts to promote data and tool sharing on various public platforms. On the other hand, we also identified a few areas that are currently facing important challenges and limit the use of CRDC resources. Table 2 summarizes the strengths and challenges of using CRDC resources from the included papers.

**TABLE 2.** Strengths and Challenges Identified From the Included Papers Regarding the Use of CRDC Resources

Strengths of Using CRDC From Current Studies	Challenges and Limitations of Using CRDC From Current Studies
Easily accessible Integrated large data sets from multiple studies Results can be easily replicable, validated, and shared through CRDC platforms	Insufficient use of multimodal data sets Insufficient use of cloud computing Need for novel ways of data analysis Need for a unified portal for easy search and analyzing data across different data commons

Abbreviation: CRDC, Cancer Research Data Commons.

In general, there are insufficient use of multimodal data sets and cloud computing and the use of new analytical methods. Most publications included in this review conducted their analyses in local computing environments, with only a few studies using CRDC's cloud computing resources despite a growing trend. Most research focused on genomics data from GDC, with other data commons and data types remaining underutilized. In addition, many studies still concentrate on descriptive or simple association analyses, highlighting a need for the adoption of more novel and advanced methodologies to facilitate easier data search and analysis across different data commons. Addressing these challenges could significantly enhance the utility and effectiveness of CRDC resources in cancer research. On the basis of these findings, we wanted to highlight a few areas for future improvements, both at CRDC's end and at the users' end.

In all publications included in this review, most of them conducted their analysis in a local computing environment. Although in an increasing trend, by far, there were only a handful of publications that used CRDC's cloud computing resources (n = 9). This highlights the need for promoting better use of cloud computing.

Leveraging cloud computing platforms in cancer research confers numerous advantages. The scalability and cost-effectiveness of cloud infrastructure allow researchers to efficiently process vast volumes of genomic and clinical data, facilitating complex analyses that were once constrained by computational limitations. In addition, researchers can securely share data sets, tools, and findings in real time, fostering a collaborative ecosystem that accelerates the pace of discovery.^37,38^

The CRDC currently has three Cloud Resources available for the public, and all three can provide user-controlled collaborative analytic environments enabling secure management and analysis of diverse data sets, including open access, controlled access, and private data. In addition to the CRDC data, there is a large collection of analytic tools and workflows available on each cloud platform, and researchers can develop and share their own tools and workflows directly in the cloud platforms as well. In the included publications, nine of them have developed and shared their applications onto CRDC's cloud platforms. For example, De Ros et al^39^ developed a web-accessible searching algorithm to identify and annotate heterogenous miRNA isoforms in SB-CGC. Nguyen et al^40^ developed an automatic multiomics pathway workflow for data preparation, dimensionality reduction, and pathway analysis. These workflows allow researchers who are not familiar with coding to prepare data and implement different analyses using SB-CGC.

CRDC's cloud platforms also have the extendibility to a wide range of commonly used bioinformatics tools, such as BigQuery, Galaxy, Jupyter notebook, RStudio, SAS, and more. These tools allow users to access publicly available analytic methods and write custom analyses in their preferred programming languages for scalable analytics. For example, Ko et al developed a classification model to distinguish glioblastoma multiforme from other forms of brain cancer using germline DNA copy number variations. The analysis was conducted in ISB-CGC environments, which allowed the authors to efficiently analyze the large volume of data and compare it with other popular machine learning models.^41^

As the field continues to evolve, cloud computing will play an increasingly important role. Through close collaboration with the cancer research community, the CRDC can ensure the availability of relevant, timely, robust, and user-friendly data and tools. The availability of easily discoverable, interoperable, and computable data is crucial for fueling both existing and emerging artificial intelligence and machine learning algorithms. These cloud resources will continue to enhance CRDC data set accessibility, enabling novel analysis techniques for unique cancer insights.

One important direction is multimodal analysis through the integration of different data types. In the era of big data, multimodal analysis is essential for managing and extracting meaningful insights from the vast amounts of information generated in cancer research, and it can facilitate cancer research in many ways.^42-45^ Understanding the interplay between molecular alterations and clinical parameters enables the identification of predictive biomarkers, refining patient stratification, and guiding personalized treatment strategies. In our review, we identified a few studies that used a multimodal approach to make predictions. For example, a recent study by Boehm et al^46^ presented a multimodal method to predict prognosis outcomes (death) in patients with ovarian cancer. In this study, a multimodal data set of patients with high-grade serous ovarian cancer was constructed, which contains patients' clinical and genomic data, as well as their histopathologic and radiologic imaging data. Using the deep learning approach, different combinations of data domains were used as predictors and the top performed model used histopathologic and radiologic data, followed by histopathologic and radiologic data plus genomic data. This example shows that the integration of multiscale clinical imaging and genomic data could increase predictivity in risk models although the clinical domain did not increase the discrimination power in this study, possibly because of the low availability of included variables.^46^

Similarly, multiomics analysis integrates data from different domains, enabling a comprehensive understanding of biologic pathways, thus facilitating the discovery of new biomarkers. For example, in a study by Guo et al,^47^ a comprehensive pan-cancer multiomics analysis was conducted to characterize the molecular features of the Notch genes. Using data from the TCGA hosted in GDC, the authors integrated multiple molecular levels data, including mRNA expression, copy number variation, methylation, and miRNA expression data. Their analysis showed the robust prognostic ability of the Notch pathway and demonstrated the potential mechanisms and cross-talks between its various RNAs and pathways.^47^

To further facilitate multimodal analysis, CRDC has made tremendous efforts in data standardization and harmonization. One such effort is the DSS, which actively harmonizes terms and ontologies and allowed field values, aiming to enhance the quality, findability, and interoperability of CRDC data. Another important tool that CRDC is currently developing is the CDA. CDA aggregates select descriptive terms about projects and data sets, combining them into unified records representing core cancer research assets like samples, subjects, and data files. This information is then presented to users as comprehensive search results, centered around key concepts of common scientific interest. Researchers can use CDA to build novel cohorts using descriptive terms such as disease name, anatomic location, race, drug used for treatment, and data type. From these results, researchers can access the data for any downstream analysis.

In addition, preliminary tools are being developed through collaboration opportunities between CRDC and other initiatives. For example, in partnership with the Advanced Research Project Agency for Health Biomedical Data Fabric Toolbox (BDF) program, CRDC will expand its capability to create a centralized platform to enhance the accessibility and usability of cancer research data. Some key features being developed include standardized data collection, privacy-preserving technology, and user-friendly dashboards for data submission and discovery. Currently, as we discussed in this review, cancer data are fragmented across multiple repositories with varying standards, making it difficult to aggregate and analyze. The BDF program addresses these issues by automating data harmonization and providing a unified interface for data access, eventually expediting cancer research by making data more accessible and usable and thus benefiting the broader biomedical community and potentially affecting other areas of disease research. The success of this collaborative program will lower barriers to data access and analysis, thereby accelerating cancer research and improving outcomes.

As CRDC continues to grow, more and more data are being added. Analyzing these increasingly diverse data sets presents significant challenges in downstream analysis; therefore, the use of novel analytical approaches will be critical.

One such method is federated learning, which trains models on heterogeneous and disparate data sets across different institutions or studies.^48^ For example, in a recent study, Terrail et al^49^ used federated learning leveraging imaging and clinical data to predict the histologic response to neoadjuvant chemotherapy for patients with early triple-negative breast cancer, whereas in another large collaborative effort, a network of International researchers developed a federated learning approach that could detect tumor boundaries for neurosurgical and radiotherapy planning in patients with glioblastoma.^50^

**FIG 4.**
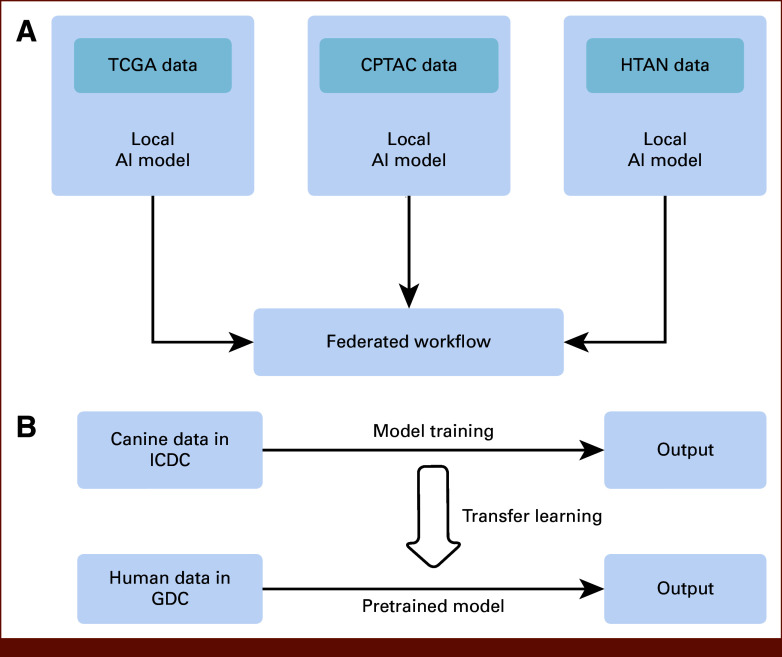
Hypothetical examples of analysis pipeline using CRDC data and federated learning (A) and transfer learning (B) methods. AI, artificial intelligence; CPTAC, Clinical Proteomic Tumor Analysis Consortium; CRDC, Cancer Research Data Commons; GDC, Genomic Data Commons; HTAN, Human Tumor Atlas Network; ICDC, Integrated Canine Data Commons; TCGA, The Cancer Genome Atlas.

In CRDC Data Commons, the abundance of data is often collected from different studies and clinical centers and many studies usually only possess insights into specific cancer types. Federated learning can address the challenge of data heterogeneity by enabling collaborative analysis without the need to centralize raw genomic information. For example, as described in Figure 4A, consider data from multiple research initiatives like TCGA, Clinical Proteomic Tumor Analysis Consortium, and Human Tumor Atlas Network, each generating genomic data specific to a particular type of cancer (eg, prostate cancer). Instead of pooling all the raw data into a central repository, a federated learning approach allows models to be trained separately on each data set within its original location. Hence, the model learns the distinct genomic signatures of prostate cancer from the data in each study without sharing the raw data itself. Once these local models are trained, their parameters or learned insights are aggregated centrally. This aggregation results in a comprehensive model that reflects the diverse genetic landscapes of prostate cancer across multiple data sets. By using federated learning, we can collaboratively analyze disparate cancer genomics data sets while maintaining data privacy and security. This methodology offers a novel way to integrate insights across studies, fostering a global understanding of cancer mechanisms and enhancing the development of precision medicine and personalized cancer therapies. The approach also bridges the gap between isolated data sets, making it a robust tool for advancing cancer research in a more inclusive and collaborative manner.

Another potentially useful machine learning method is transfer learning. Transfer learning emerges as a potent strategy in advancing cancer genomics studies in recent years, particularly when confronted with the challenge of integrating information from diverse data sets. For example, Hao et al^51^ developed a transfer learning–based model for brain tumor classification, and their model was able to achieve good performance and reduce annotation costs. In the context of data from CRDC, unique genomic data sets were compiled from different studies, and transfer learning would allow for the extraction of knowledge learned from one data set and applying it to another. This method leverages pretrained models on a source data set and transfers the learned features to a target data set, aiding in the analysis of genomics data from different sources. Consider the following scenario (Fig 4B): the ICDC hosts extensive data from studies on the genomic landscape of canine brain cancer, leveraging data from projects like the Comparative Oncology Program and others. A transfer learning approach can be used to harness the knowledge extracted from these studies, such as the identification of genetic markers associated with brain cancer or the efficacy of drug responses across different cancer subtypes. Alternatively, a predictive model for brain cancer risk could be trained using the rich data set from the ICDC.

In transfer learning, the knowledge or the pretrained model developed from the canine data can be transferred and fine-tuned for application in a different, yet related, domain—such as human brain cancer data from the GDC. Methodologically, this involves initializing a new model with the parameters from the ICDC-trained model and then fine-tuning this model using the human data. This approach allows the model to retain valuable insights from the canine data while adapting to the specifics of the human data set, subsequently enhancing the efficiency of analyzing new genomic data.

Finally, CRDC's resources are also ideal for validation studies. Validation studies play a pivotal role in enhancing the robustness and generalizability of cancer genomics studies when extending analyses across different data sets. These studies involve the independent assessment of findings, often derived from one data set, on a distinct data set to confirm the consistency and validity of observed genomic patterns. In the realm of cancer omics, where heterogeneity is inherent, validation studies provide a critical step toward ensuring the reliability of identified biomarkers, genetic signatures, or treatment responses. These validation studies aim to replicate or compare the observed findings using different data sets and, therefore, serve as a crucial step in the translational journey from discovery to application in cancer research. They mitigate the risk of false positives, enhance the reproducibility of findings, and contribute to the establishment of robust findings with potential clinical implications.

This review has certain limitations that should be acknowledged. First, by focusing explicitly on studies that directly reference CRDC resources, we confined our search to the current names of CRDC's individual components. This might have led to the exclusion of relevant studies that used CRDC resources but did not explicitly cite them under these specific terms. Therefore, we recommend the CRDC developing and publishing a standardized citation format for CRDC resources, which could improve the identification and inclusion of relevant studies. Second, as this is a scoping review, we did not assess the risk of bias or the validity of the included studies. While this aligns with our primary goal of understanding the broad impact of CRDC on cancer research, it may limit the depth of our conclusions. Finally, the rapidly evolving nature of both cancer research and the CRDC infrastructure means that some of the studies included may not fully reflect the current capabilities or utilization of the CRDC, potentially limiting the review's timeliness.

In conclusion, in this scoping review of the current literature, we examined how NCI CRDC resources are currently used, with a close look at the trends and utilization patterns. As our findings suggest, the CRDC has served as a foundation for fostering cancer research. We also identified a few areas of interest for future research, such as better use of cloud computing and heterogeneous data sets and the use of sophisticated analytical methods. In summary, CRDC, as the foundation of a National Cancer Data Ecosystem, will continue empowering the research community to effectively leverage cancer-related data, uncover novel strategies, and address the needs of cancer patients, ultimately combatting this disease more effectively.
